# The circulating ANGPTL8 levels show differences among novel subgroups of adult patients with diabetes and are associated with mortality in the subsequent 5 years

**DOI:** 10.1038/s41598-020-69091-y

**Published:** 2020-07-30

**Authors:** Huajie Zou, Wu Duan, Zeqing Zhang, Xi Chen, Puhan Lu, Xuefeng Yu

**Affiliations:** 1 Division of Endocrinology, Department of Internal Medicine, Tongji Hospital, Tongji Medical College, Huazhong University of Science and Technology, 1095 Jiefang Avenue, Wuhan, 430030 China; 2 Division of Endocrinology, Department of Internal Medicine, Qilu Hospital of Shandong University, Jinan, China

**Keywords:** Diabetes, Risk factors

## Abstract

ANGPTL8, an important regulator of glucose and lipid metabolism, is associated with diabetes, but the role of ANGPTL8 in the outcomes of novel subgroups of diabetes remains unclear. To assess the circulating ANGPTL8 levels in novel subgroups of diabetes and their association with health outcomes, we performed a data-driven cluster analysis (k-means) of patients with newly diagnosed diabetes (741 patients enrolled from 2011 through 2016) from the Risk Evaluation of Cancers in Chinese Diabetic Individuals: a longitudinal (REACTION) study. The primary outcomes were mortality from all causes and cardiovascular diseases (CVD), and the secondary outcome was any cardiovascular event. Comparisons among groups were performed using the Kruskal–Wallis test, and the correlations between variables were assessed using the Pearson correlation test. Logistic regression was used to detect associations between the risk of outcomes and the ANGPTL8 levels. We identified four replicable clusters of patients with diabetes that exhibited significantly different patient characteristics and risks of all-cause mortality. The serum ANGPTL8 levels in the cluster of mild age-related diabetes (MARD), severe insulin-resistant diabetes (SIRD), and severe insulin-deficient diabetes (SIDD) were significantly higher than those in the mild obesity-related diabetes (MOD) cluster (685.01 ± 24.50 vs. 533.5 ± 18.39, *p* < 0.001; 649.69 ± 55.83 vs. 533.5 ± 18.39, = 0.040; 643.29 ± 30.89 vs. 533.5 ± 18.39, *p* = 0.001). High circulating ANGPTL8 levels were more highly associated with a greater hazard of all-cause mortality (quartile 4 vs 1: risk ratio [RR] 3.23, 95% CI 1.13–9.22; per unit increase in the Z score: RR 1.53, 95% CI 1.17–2.01) than low circulating ANGPTL8 levels. In conclusion, this 5-year follow-up REACTION study revealed that the circulating ANGPTL8 levels show differences among novel subgroups of adult patients with diabetes and are associated with all-cause mortality in the subsequent 5 years.

## Introduction

Angiopoietin-like protein 8 (ANGPTL8), which is also known as betatrophin, TD26, “refeeding induced in fat and liver” (RIFL) and lipasin, is a novel hormone and potentially a potent stimulator of β-cell proliferation^1–5^. However, this finding was questioned by other studies that showed that mice lacking ANGPTL8 exhibit normal glucose and insulin tolerance^2^. Subsequent studies revealed that ANGPTL8 closely interacts with and controls ANGTPL3/4 by binding to lipoprotein lipase (LPL) and regulating triglyceride (TG) metabolism^6–9^. Moreover, ANGPTL8 has been described as a novel target gene of the vitamin D receptor involved in non-alcoholic fatty liver pathogenesis^10^. These findings indicate that ANGPTL8 plays critical roles in the development of various metabolic diseases^11,12^. Diabetes, which is closely related to glucose and lipid metabolism disorders, is the fastest increasing disease worldwide and poses a substantial threat to human health^13^. Epidemiological studies have also demonstrated that the ANGPTL8 levels are increased in patients with long-standing type 1 diabetes mellitus (T1DM)^14^ and type 2 diabetes mellitus (T2DM)^15–17^, but contradictory results have been obtained in other studies^18,19^. The currently available evidence does not support the direct effects of ANGPTL8 on glucose and lipid metabolism^1,16,20–23^, and one explanation for these controversial results could be that the classification of diabetes is not sufficiently precise.

Emma Ahlqvist and colleagues used six variables to identify five exclusive subgroups of diabetes, which were verified by distinct progression trajectories of microvascular complications^24^. The novel diabetes subgroups have also been validated in the Chinese population^25^. The physiological basis of the features characterizing each cluster of novel diabetes subgroups provides a strong rationale for investigating the genetic and molecular mechanisms that lead to the observed heterogeneity in the presentation and progression of diabetes in adults^26^. Based on these findings, the present study aimed to evaluate the levels of circulating ANGPTL8, which serves as an important factor in glucose and lipid metabolism, in subjects belonging to different novel diabetes subgroups and its association with subsequent events or complications.

## Methods

### Study population

The participants in the present study were newly diagnosed with diabetes and recruited from Hubei Province of China from 2011 to 2012 as part of the Risk Evaluation of Cancer in Chinese Diabetic Individuals: a longitudinal (REACTION) study, which was included 259,657 adults aged at least 40 years in 25 communities across mainland China^27^. The Committee on Human Research at Tongji Hospital, Tongji Medical College, Huazhong University of Science and Technology, approved the study protocol, and all the participants provided written informed consent. All the methods were in accordance with the relevant guidelines and regulations. The vital status of the cohort members was determined from 2011 to 2012 through December 31, 2016. We confirmed that all the methods agreed with the relevant guidelines and regulations. Our present study was performed in accordance with the STROBE statement.

### Study outcomes

The primary outcomes were mortality from all causes and cardiovascular diseases (CVDs). The secondary outcome was any cardiovascular event [a composite of heart failure (HF), stroke, and myocardial infarction (MI)]. All these outcomes were confirmed by death certificates and hospital records.

### Clinical and biochemical evaluation

As previously described in the REACTION study^27^, information on sociodemographic characteristics, lifestyle factors, medical history and family history was collected by trained staff using a standard questionnaire. All the participants were asked to fast for at least 10 h prior to undergoing the oral glucose tolerance test (OGTT), and blood samples were obtained from all the participants for the analysis of various biochemical parameters.

### Assessment of diabetes and insulin resistance

The diagnosis of T2DM was based on the diagnostic criteria established by the American Diabetes Association in 2009^28^: Specifically, a diagnosis of T2DM was made if one of the following conditions were met: fasting plasma glucose (FPG) ≥ 7.0 mmol/l or 2-h postprandial blood glucose (2hPG) ≥ 11.1 mmol/l. The β cell function was assessed through the homeostasis model assessment of β cell function (HOMA-β)^29^, and insulin resistance was estimated by the homeostasis model assessment of insulin resistance (HOMA-IR)^29^.

### Measurement of ANGPTL8

The fasting serum ANGPTL8 levels were assessed using ELISA kits (EIAab Science, Wuhan, China; Catalogue No. E11644 h) in accordance with the manufacturer’s instructions; this kit has an intra-assay CV < 6.5% and an inter-assay CV < 9.2% (provided by the manufacturer). All the samples were analysed in duplicate.

### Cluster analysis

We used k-means analysis to cluster the data into four groups according to five variables: age at diagnosis, body mass index (BMI), HbA1c, HOMA-β and HOMA-IR^24^. Men and women were clustered separately to avoid stratification due to sex-dependent differences in the cluster variables and to provide separate cohorts for validation of the results. K-means clustering was performed in TensorFlow using a k value of 4 and the k-means runs function (runs = 100). T-distributed stochastic neighbour embedding (t-SNE) was used to visualize the four clusters in 3D^30^.

### Statistical analysis

The baseline characteristics of the participants are presented as the means ± SEMs for continuous variables and numbers (proportions) for categorical variables. The normality of the distribution of the data was tested using the Kolmogorov–Smirnov test^16^. The variables that were not normally distributed were compared among the groups using a nonparametric test followed by the Kruskal–Wallis test. The correlations among variables were assessed using the Pearson correlation test. We also compared the ANGPTL8 levels among groups by ANCOVA after adjustment for age based on the results from the Pearson correlation test. A binary logistic regression analysis was conducted to calculate the risk ratios (RRs) and 95% confidence intervals (95% CIs) for outcomes in the various quartiles of ANGPTL8 and each 1-unit change in the Z score of ANGPTL8. Receiver-operator characteristic (ROC) curves were drawn, and the areas under the curve (AUCs) for ANGPTL8, BMI, HbA1c, HOMA-IR, TG and total cholesterol with respect to mortality and CVD events were calculated. A two-tailed *p* value < 0.05 was considered to indicate significance. SPSS version 20.0 was used for all the analyses.

### Ethics approval and consent to participate

The Committee on Human Research at Tongji Hospital, Tongji Medical College, Huazhong University of Science and Technology, approved the study protocol, and all the participants provided written informed consent.

## Results

### Characteristics of the study population

Among the 811 patients with diabetes enrolled in the study, patients with CVD (n = 69) were excluded. As shown in Table 1, the mild age-related diabetes (MARD) cluster, which comprised 294 (39.7%) patients, was older than the other clusters and characterized by a relatively low BMI, modest metabolic derangements and the highest mortality (11.6%). The mild obesity-related diabetes (MOD) cluster, which included 281 (37.9%) patients, was characterized by a low age at onset and presented the highest values for BMI, average blood glucose, β-cell function, and insulin resistance. The severe insulin-resistant diabetes (SIRD) cluster, which consisted of 51 (6.9%) patients, was characterized by insulin resistance (high HOMA-IR index) and a high BMI. The severe insulin-deficient diabetes (SIDD) cluster, which comprised 115 (15.5% of total 741 patients) patients, was characterized by the lowest insulin secretion (low HOMA-β index) and poor metabolic control. The patient distribution and cluster characteristics are also shown in Figure S1. The visualization of the clusters showed that all the patients were well separated into the four clusters (Appendix Video 1).

**Table 1:** Clinical and metabolic parameters for subjects in novel subgroups of adult diabetes.

Characteristics	MARD	MOD	SIRD	SIDD	All	p value
N (%)	294 (39.7)	281 (37.9)	51 (6.89)	115 (15.5)	741	
Age (years)	67 ± 0.47	53 ± 0.39	58 ± 1.57	54 ± 0.80	59 ± 0.38	< 0.001
Male sex—no. (%)	136 (46.3)	61 (21.7)	23 (3.3)	41 (35.7)	261 (35.2)	< 0.001
BMI (kg/m^2^)	22 ± 0.15	26 ± 0.20	24 ± 0.41	24 ± 0.32	24 ± 0.13	< 0.001
HbA1c (%)	6.19 ± 0.05	6.73 ±	6.38 ± 0.19	10.74 ± 0.18	7.12 ± 0.07	< 0.001
HOMA-IR	2.02 ± 0.11	2.99 ± 0.12	14.02 ± 0.87	3.50 ± 0.20	3.35 ± 0.14	< 0.001
HOMA-β (%)	36.81 ± 1.54	48.56 ± 1.90	178.16 ± 18.60	16.08 ± 1.18	47.78 ± 2.08	< 0.001
HDL (mmol/l)	1.82 ± 0.05	1.57 ± 0.04	1.69 ± 0.10	1.87 ± 0.10	1.72 ± 0.03	< 0.001
LDL (mmol/l)	2.91 ± 0.05	3.05 ± 0.05	2.64 ± 0.11	3.19 ± 0.09	2.99 ± 0.03	< 0.001
Total cholesterol (mmol/l)	5.17 ± 0.06	5.27 ± 0.06	4.85 ± 0.90	5.42 ± 0.11	5.22 ± 0.04	0.008
TG (mmol/l)	1.56 ± 0.07	2.07 ± 0.11	2.10 ± 0.24	1.94 ± 0.12	1.85 ± 0.06	< 0.001
ANGPTL8 (pg/ml)	685.01 ± 24.50	533.5 ± 18.39	649.69 ± 55.83	643.29 ± 30.89	618.67 ± 13.65	< 0.001
Outcomes—no. (%)						
Death	34 (11.6)	4 (1.4)	0 (0)	8 (7.0)	46 (6.2)	< 0.001
MI	3 (1.0)	3 (1.0)	0 (0)	2 (1.8)	8 (1.1)	0.793
Stroke	8 (2.7)	5 (1. 8)	0 (0)	2 (1.7)	15 (2.0)	0.592
HF	2 (0.7)	2 (0.7)	4 (7.8)	1 (0.9)	9 (1.2)	< 0.001

*BMI* body-mass index, *HOMA-IR* homeostasis model assessment of insulin resistance, *HOMA-*β homeostasis model assessment of β cell function, *HDL* high density lipoprotein, *LDL* low density lipoprotein, *TG* triglycerides, *MI* myocardial infarction, *HF* heart failure, *MARD* mild age-related diabetes, *MOD* mild obesity-related diabetes, *SAID* severe autoimmune diabetes, *SIDD* severe insulin-deficient diabetes, *SIRD* severe insulin-resistant diabetes.

### Association between ANGPTL8 and novel diabetes

As shown in Table 1, the serum ANGPTL8 levels were significantly elevated in the MARD, SIRD and SIDD clusters compared with the MOD cluster (685.01 ± 24.50 vs. 533.5 ± 18.39, *p* < 0.001; 649.69 ± 55.83 vs. 533.5 ± 18.39, *p* = 0.040; 643.29 ± 30.89 vs. 533.5 ± 18.39, *p* = 0.001). However, no significant differences in the ANGPRL8 levels were found among the MARD, isolated SIRD and isolated SIDD clusters (all *p* values > 0.05). Consistently, the MARD cluster presented higher mortality than the SIRD, SIDD and MOD clusters (*p* < 0.05).

### Association between ANGPTL8 and metabolic indexes

We subsequently studied the correlations between the ANGPTL8 levels and various metabolic variables, including age, BMI, HbA1c, HOMA-IR, HOMA-β and lipid profiles, in all the participants through a Pearson correlation analysis. After controlling for multiple variables, the ANGPTL8 levels were positively correlated with age (r = 0.169, *p* < 0.001; Model 3 in Table S1), FPG (r = 0.088, *p* = 0.018) and TG (r = 0.129, *p* < 0.001). However, the negative correlation of ANGPTL8 with high-density lipoprotein (HDL) (*r* = −0.093, p = 0.01, Model 2) and low-density lipoprotein (LDL) (*r* = −0.076, *p* = 0.04, Model 2) was diminished after adjusting for other lipid profiles (*p* values > 0.05, Model 3). No association was found between ANGPTL8 and BMI, HbA1c, fasting insulin, HOMA-IR, HOMA-β and total cholesterol (all *p* values > 0.05). ANCOVAs with age as a covariate were performed to control for this potentially confounding effect. As shown in Fig. S2, differences in the ANGPTL8 levels were still found between the MARD and SIDD clusters and the MOD cluster correcting for age (MARD vs. MOD: *p* = 0.0042; SIDD vs. MOD, *p* = 0.011), but no significant differences in the ANGPRL8 levels were found between the SIRD and MOD patients after adjusting for age.

### ANGPTL8 and outcomes

The subjects were divided into four groups based on the ANGPTL8 quartiles. After adjusting for multiple factors, such as age, gender, lipid profile, HbA1c and HOMA-IR, the patients in the highest quartile of ANGPTL8 presented a more than threefold higher risk of death (RR 3.23; 95% CI 1.13–9.22; Table 2). After Z-transform standardization, the RR for all-cause mortality was 1.53 (95% CI 1.17–2.01) per unit increase in the Z score of ANGPTL8. In the crude model, the participants within the highest ANGPTL8 quartile had RRs for CVD mortality and CVD events of 1.49 (95% CI 1.11–1.99) and 1.31 (95% CI 1.04–1.66), respectively, compared with the participants within the lowest ANGPTL8 quartile. A similar pattern of joint association was observed after Z-transform standardization. However, no significant association was found between ANGPTL8 and rates of CVD mortality and CVD events in the fully adjusted model.

**Table 2:** Risk ratios for mortality and CVD event according to categories of ANGPTL8.

Outcome	Model 1	Model 2	Model 3	Model 4
**Mortality**				
All-cause mortality				
Q1 (reference)	1	1	1	1
Q2 (RR, 95% CI)	1.63 (0.52–5.07)	1.79 (0.55–5.82)	1.53 (0.45–5.15)	1.44 (0.42–4.89)
Q3 (RR, 95% CI)	2.27 (0.78–6.69)	1.90 (0.63–5.78)	1.82 (0.59–5.60)	1.74 (0.56–5.40)
Q4 (RR, 95% CI)	4.86 (1.78–13.13)	3.23 (1.15–9.10)	3.28 (1.15–9.34)	3.23 (1.13–9.22)
Per unit increase in Z score	1.66 (1.31–2.11)	1.45 (1.12–1.87)	1.51 (1.16–1.98)	1.53 (1.17–2.01)
CVD mortality				
Q1 (reference)	1	1	1	1
Q2 (RR, 95% CI)	1.78 (0.51–6.19)	1.99 (0.55–7.26)	1.64 (0.43–6.37)	1.50 (0.38–5.83)
Q3 (RR, 95% CI)	1.78 (0.51–6.19)	1.48 (0.41–5.33)	1.47 (0.40–5.43)	1.38 (0.37–5.15)
Q4 (RR, 95% CI)	3.14 (0.99–9.92)	2.03 (0.61–6.72)	2.14 (0.63–7.19)	2.08 (0.62–7.05)
Per unit increase in Z score	1.49 (1.11–1.99)	1.28 (0.93–1.76)	1.36 (0.98–1.90)	1.38 (0.99–1.93)
CVD event				
Q1 (reference)	1	1	1	1
Q2 (RR, 95% CI)	0.85 (0.38–1.89)	0.85 (0.37–1.94)	0.79 (0.34–1.85)	0.77 (033–1.80)
Q3 (RR, 95% CI)	0.77 (0.34–1.75)	0.64 (0.28–1.49)	0.68 (0.29–1.58)	0.64 (0.27–1.51)
Q4 (RR, 95% CI)	1.56 (0.77–3.18)	1.07 (0.50–2.26)	1.14 (0.53–2.44)	1.11 (0.52–2.39)
Per unit increase in Z score	1.31 (1.04–1.66)	1.16 (0.90–1.49)	1.22 (0.94–1.58)	1.22 (0.94–1.58)

Model 1 was unadjusted.

Model 2 was adjusted for age, sex and BMI.

Model 3 was adjusted for all variables in model 2 plus HDL, LDL, TG and total cholesterol.

Model 4 was adjusted for all variables in model 2 plus HDL, LDL, TG and total cholesterol, HbA1c, HOMA-IR.

*CVD* cardiovascular disease, *BMI* body-mass index, *HOMA-IR* homeostasis model assessment of insulin resistance, *HOMA-*β homeostasis model assessment of β cell function, *HDL* high density lipoprotein, *LDL* low density lipoprotein, *TG* triglycerides.

A comparison of the ANGPTL8, BMI, HbA1c, HOMA-IR, TG and total cholesterol for predicting 5-year mortality and CVD events was conducted through a ROC curve analysis. The ANGPTL8 cut-off for predicting all-cause mortality was higher than 521.25 pg/ml. The AUC value obtained for ANGPTL8 was 0.667 (95% CI 0.584–0.751), which was higher than those found for BMI, HbA1c, HOMA-IR, TG and total cholesterol. If the ANGPTL8 was higher than 521.25, the sensitivity was 78.3%, and the specificity was 50.0%. Similarly, the AUC values of ANGPTL8 for predicting CVD mortality and CVD events were higher than those found for BMI, HbA1c, HOMA-IR, TG and total cholesterol (Fig. S3).

## Discussion

In this population-based longitudinal study of novel clusters of diabetes, we found that ANGPTL8 was significantly elevated in the MARD, SIRD, and SIDD clusters compared with the MOD cluster. ANGPTL8, which is a novel protein that has been proposed to serve as an important regulator of glucose and lipid metabolism, might play an important role in the progression of diabetes. Previous studies have yielded controversial results regarding the association between ANGPTL8 and diabetes^15,16,19,31^. One explanation for these controversial results might be that the classification of diabetes is not sufficiently precise. We thus attempted to clarify the circulating ANGPTL8 levels in novel subgroups of diabetes proposed by Emma Ahlqvist^24^ and the association of the ANGPTL8 levels with outcomes in the subsequent 5 years. First, we found that ANGPTL8 was relatively low in the novel MOD cluster of diabetes. Further analysis suggested that the ANGPTL8 levels were positively correlated with age and TG but negatively correlated with HDL. No association was found for ANGPTL8 with BMI, HOMA-IR or HOMA-β. Therefore, the relatively low level of ANGPTL8 in the MOD cluster might be due to the low age at diabetes onset. Furthermore, several cross-sectional studies have also suggested that the circulating ANGPTL8 levels in obese patients are lower than those in participants with a normal weight^18,32,33^. According to Guo^34^, the excessive secretion of adipokines might be a central player in the pathophysiologies of diabetes mellitus, IR, dyslipidaemia, and atherosclerosis in obesity. In the obese state, the ANGPTL8 levels might change in response to certain factors as a compensatory mechanism aimed at maintaining a certain balance. Therefore, the level of ANGPTL8 might not change or decrease in obesity^34^. The null association between ANGPTL8 and HOMA-IR or HOMA-β was consistent with the results obtained in a previous study^35^, which found that the ANGPTL8 levels in patients with type 2 diabetes are not correlated with blood glucose or insulin resistance. Second, the elevated ANGPTL8 levels found in the SIDD, SIRD and MARD clusters compared with the MOD cluster might be due to higher FPG in SIDD, higher TG in SIRD, and old age in MARD. These factors are closely associated with atherosclerosis^36^, which might provide some clues for the heterogeneity in the CVD outcomes in diabetes.

Emma Ahlqvist et al. also demonstrated that the novel subgroups of diabetes might provide some information on subsequent events or complications in the different clusters. These researchers found that the risk of kidney complications was substantially increased in patients with SIRD^24^. We thus further analysed the outcomes in the novel subgroups. The results suggested that patients with MARD were associated with an increased risk for all-cause mortality. However, no difference in the risks for CVD and CVD mortality were found in the four clusters. Interestingly, the patients belonging to the MARD cluster also presented the highest ANGPTL8 level. We therefore performed further analyses and found that the increased risk for all-cause mortality was associated with ANGPTL8, even after adjustment for gender, sex and BMI, lipid profile, HbA1c and HOMA-IR. This finding suggests that ANGPTL8 might play an important role in the progression of health outcomes due to diabetes. Previous studies have implied that ANGPTL8 might contribute to the progression of CVD^37,38^. Unfortunately, we did not detect an association between ANGPTL8 and CVD and CVD-related mortality in the fully adjusted model, which might be due to the limited events and duration of our study. However, ANGPTL8 better predicts the risk for all-cause mortality, CVD-related mortality and CVD events compared with several traditional risk factors for CVD in diabetes, such as BMI, HbA1c, HOMA-IR, TG and total cholesterol.

The underlying mechanism driving the observed differences in the ANGPTL8 levels among novel diabetes groups and the association of ANGPTL8 with detrimental outcomes also remain unclear. ANGPTL8 is a secreted inhibitor of lipoprotein lipase, which is a key enzyme in plasma triglyceride metabolism^9^. Previous studies have also demonstrated that ANGPTL8 is closely related to inflammation^39–42^, which is increasingly considered a pathologic mediator of CVD, diabetes and its complications^43^. We also found that ANGPTL8 is positively correlated with TG but negatively correlated with HDL which implies that ANGPTL8 might detrimentally contribute to health by regulating lipid metabolism. Several studies have shown that ANGPTL8 is related to HDL-C dysfunction^1,44,45^ and future changes in the TG levels^46^. ANGPTL8 is also involved in the association between dyslipidaemia and arteriosclerosis^47^, regardless of glucose intolerance or diabetes mellitus^33,48^. ANGPTL8 exerts a negative effect on the HDL-mediated cholesterol efflux capacity^44^ and a strong link to subclinical atherosclerosis^47^, and its levels are significantly increased in patients with coronary disease proportionally to the disease severity^49^. Genetic studies and investigations of molecular pathways have revealed that miR-143-3p regulates the ANGPTL8 transcript and protein expression levels^50^, that the prevalence of T2DM and impaired glucose tolerance is greater in subjects with the R59W ANGPTL8 variant^51^, and that the concomitant presence of CETP B1, NOS3 T and ANGPTL8 T alleles augments the risk of CVD and T2DM^37^. Furthermore, excess adiposity might lead to cancer development through dysfunctional adipose tissues and altered signalling pathways^52,53^. Therefore, the role of ANGPTL8 in lipid metabolism might also contribute to cancer or other detrimental outcomes. Unfortunately, we cannot perform a further analysis on cancer due to the limited cancer events in our study. Therefore, the difference in the ANGPTL8 levels among the novel diabetes groups might be due to their correlation with the metabolic characteristics in each cluster, such as higher FPG in SIDD, higher TG in SIRD, and old age in MARD, and could further lead to a detrimental outcome.

This study is the first longitudinal study to detect the association between ANGPTL8 and outcomes of diabetes, particularly those in novel subgroups of diabetes. The limitations of this study include its observational design and low or infrequent counts of some outcomes. Therefore, we cannot perform further analysis of the association between ANGPTL8 and outcomes in each cluster due to the limited number of events. Furthermore, we did not assess the glutamic acid decarboxylase antibodies (GADAs) in the patients. According to Zou et al.^25^ patients with GADA-positive diabetes are usually diagnosed prior to their detection through screening due to acute diabetes complications; thus, the prevalence of GADA-positive type 2 diabetes is 5.9% in China and 4.7% in the USA, and lower values might even be obtained through population-based screening. It is thus difficult to detect GADA-positive diabetes.

## Conclusion

In conclusion, this 5-year follow-up study of the REACTION study revealed that the circulating ANGPTL8 levels show differences among novel subgroups of adult patients with diabetes and are associated with all-cause mortality in the subsequent 5 years.

## Supplementary information

Supplementary Video 1.

Supplementary Information.
