# Supplementary material for: The circulating ANGPTL8 levels show differences among novel subgroups of adult patients with diabetes and are associated with mortality in the subsequent 5 years
Source: Sci Rep. 2020 Jul 30;10:12859. doi: 10.1038/s41598-020-69091-y (PMC7393150; doi:10.1038/s41598-020-69091-y)
Supplement: Supplementary file 2 — Supplementary Information. [file 41598_2020_69091_MOESM2_ESM.pdf]

Circulating ANGPTL8 levels differ in novel subgroups of adult patients with diabetes and are associated with mortality in subsequent 5 years

Huajie Zou[1], Wu Duan[1,2], Zeqing Zhang[1], Chen Xi[1], Puhan Lu[1], Xuefeng Yu[1]*

1. Division of Endocrinology, Department of Internal Medicine, Tongji Hospital, Tongji Medical College, Huazhong University of Science and Technology, Wuhan, China.

2. Division of Endocrinology, Department of Internal Medicine, Qilu Hospital of Shandong University

*__Correspondence__:

Xuefeng Yu, M.D., Ph.D., Professor of Medicine, Division of Endocrinology, Department of Internal Medicine, Tongji Hospital, Tongji Medical College, Huazhong University of Science and Technology, 1095 Jiefang Avenue, Wuhan 430030, China.

Email: xfyu188@163.com

Tel / Fax: +86 027 83662883.

Table S1. Partial correlations between ANGPTL8 levels and clinical variables in all study participants.

	ANGPTL8		ANGPTL8 (age, sex, and BMI adjusted) *		ANGPTL8 (age, sex, and BMI, HDL, LDL, cholesterol and TG adjusted) *	
	r	*p* value	Partial r	*p* value	Partial r	*p* value
Age	0.186	0.000	0.153	0.000	0.169	0.000
BMI	-0.087	0.018	-0.019	0.605	-0.035	0.341
FPG	0.130	0.728	0.081	0.029	0.088	0.018
INS	-0.019	0.607	0.015	0.675	-0.009	0.815
HbA1c	-0.020	0.581	0.057	0.123	0.061	0.102
HOMA-IR	-0.007	0.849	0.035	0.344	0.012	0.737
HOMA-β	-0.038	0.300	-0.014	0.695	-0.028	0.445
HDL	-0.066	0.071	-0.093	0.011	-0.057	0.121
LDL	-0.095	0.009	-0.076	0.041	0.000	0.990
Total cholesterol	-0.042	0.259	-0.021	0.567	-0.029	0.435
TG	0.102	0.005	0.151	0.000	0.129	0.000

* when a variable was calculated in the Partial correlation, it would not be included in adjustment model.

Abbreviation: BMI, body-mass index; HOMA-IR, homeostasis model assessment of insulin resistance; HOMA- β, homeostasis model assessment of β cell function; HDL, high density lipoprotein; LDL, low density lipoprotein; TG, triglycerides; MI, myocardial infarction; HF, heart failure; MARD, mild age-related diabetes; MOD, mild obesity-related diabetes; SAID, severe autoimmune diabetes; SIDD, severe insulin- deficient diabetes; SIRD, severe insulin-resistant diabetes.

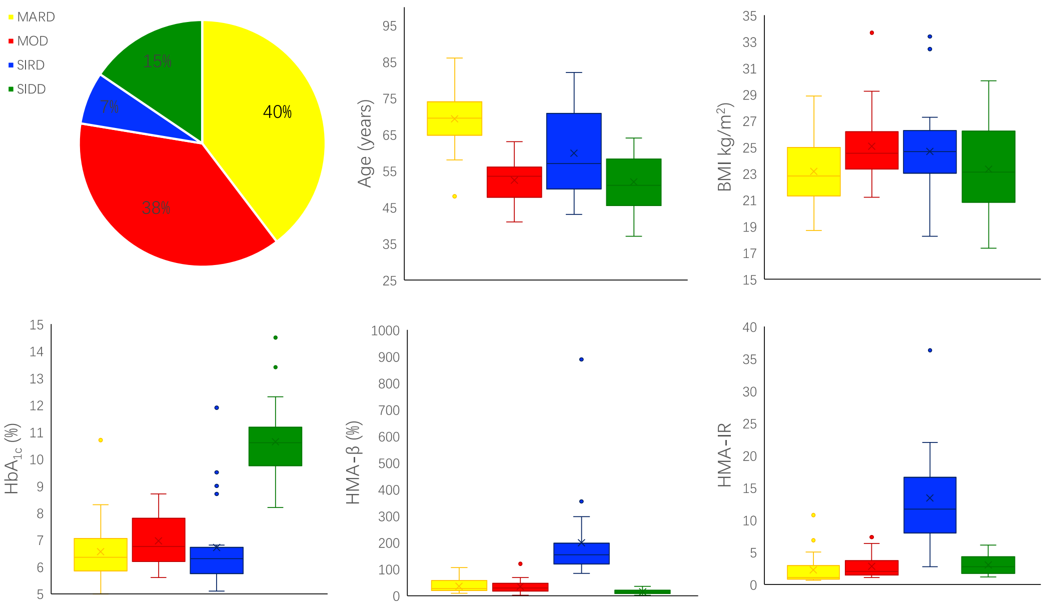

Figure S1. Participant distribution and cluster characteristics

MARD=mild age-related diabetes. MOD=mild obesity-related diabetes. SAID=severe autoimmune diabetes. SIDD=severe insulin-deficient diabetes. SIRD=severe insulin-resistant diabetes. HOMA-β=homoeostatic model assessment estimates of β-cell function. HOMA-IR=homoeostatic model assessment estimates of insulin resistance.

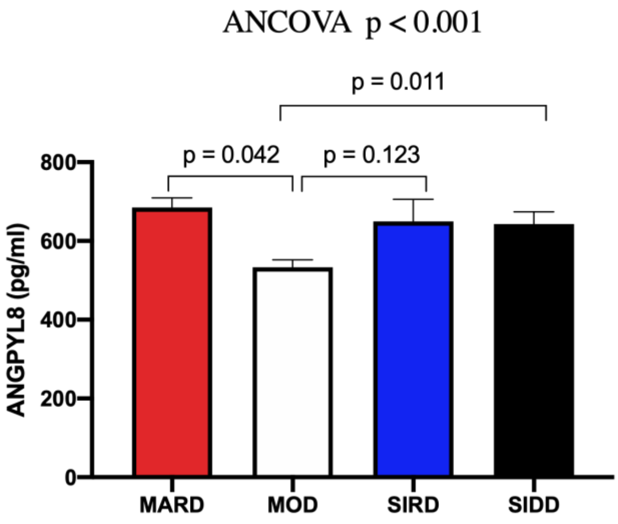

Figure S2. ANGPTL8 levels in novel diabetes groups adjusted for age.

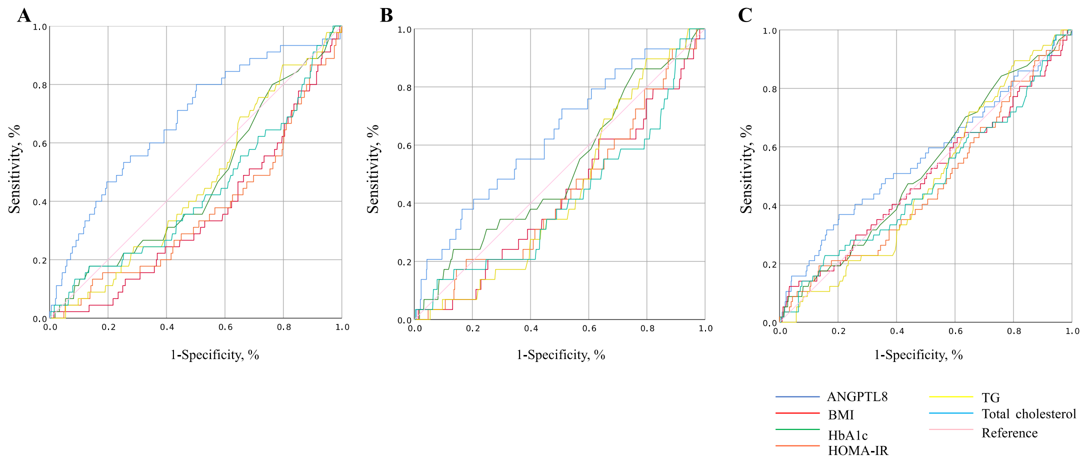

Figure S3. Comparison of areas under the ROC curve for predicting 5-year (A) all-cause mortality, (B) CVD mortality, (C) CVD events with ANGPTL8, BMI, HbA1c, HOMA-IR, TG and total cholesterol.
